# Supplementary material for: An Anti-CD7 Antibody–Drug Conjugate Target Showing Potent Antitumor Activity for T-Lymphoblastic Leukemia (T-ALL)
Source: Biomolecules. 2024 Jan 15;14(1):106. doi: 10.3390/biom14010106 (PMC10813019; doi:10.3390/biom14010106)

Figure S1. CCK-8 assay to analyze cell cytotoxicity. IC50 values were determined using curve fitting by nonlinear regression as the concentration of the drug that causes 50 % loss of cell viability compared to untreated cells. An anti-SLC3A2 ADC was used as control ADC.

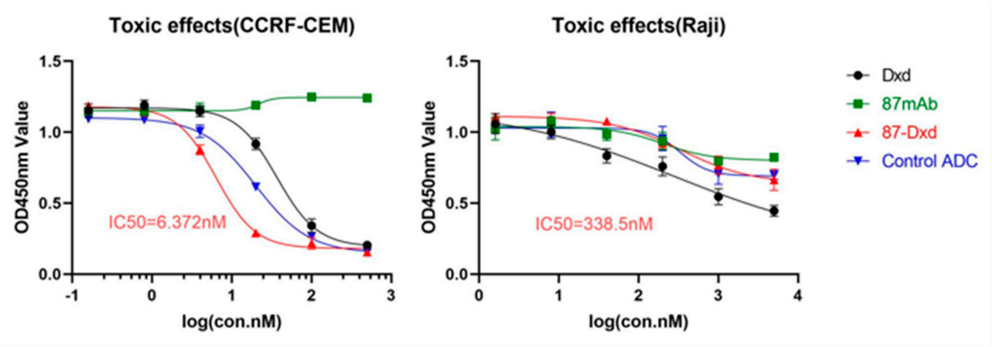

.

Figure S2. Drug-to-Antibody Ratio (DAR) Analysis of J87-Dxd. The HIC assay was carried out using a TOSOH Butyl - NPR column (35 × 4.6 mm). Samples were injected onto the column equilibrated in 100% buffer A (1.5 M ammonium sulfate, 25 mM sodium phosphate, pH 6.95) and eluted by a linear gradient to 100% B (75% 25 mM sodium phosphate, pH 6.95, 25% isopropanol) over a 30-min period with detection at 280 nm. D0 and D2 represent DAR0 and DAR2, respectively.

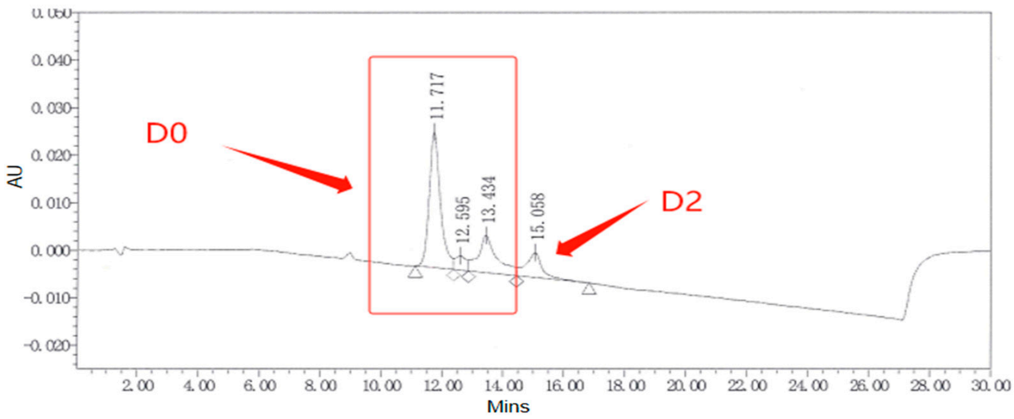

Figure S3. Overall survival of mice treated Dxd or PBS control. A total of 3 × 10^6 CCRF-CEM-luciferase cells were implanted in mice with caudal vein injection. 9 days later, CCRF-CEM mouse models were divided into two groups (n=5) and treated with Dxd (10 μg/kg, i.p.) and PBS, respectively. Mouse survival was recorded every 3 days. ns represents no significance.

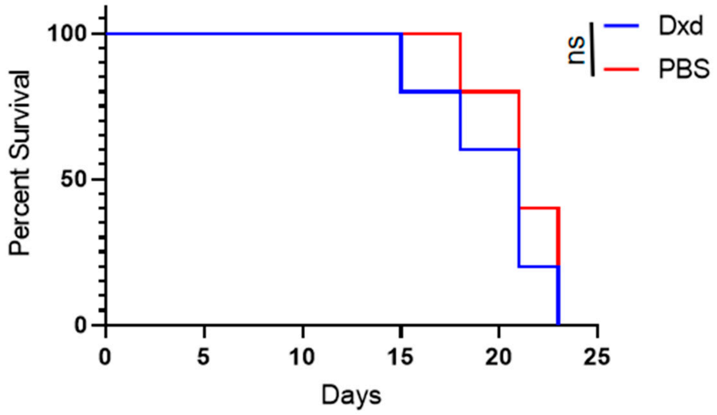

Supplement: Supplementary file 1 [file biomolecules-14-00106-s001.zip › biomolecules-2787690-supplementary.pdf]
